# Developmental Exposure to Low-Dose PBDE-99: Effects on Male Fertility and Neurobehavior in Rat Offspring

**DOI:** 10.1289/ehp.7421

**Published:** 2004-11-04

**Authors:** Sergio N. Kuriyama, Chris E. Talsness, Konstanze Grote, Ibrahim Chahoud

**Affiliations:** Institute of Clinical Pharmacology and Toxicology, Department of Toxicology, Charité University Medical School Berlin, Campus Benjamin Franklin, Berlin, Germany

**Keywords:** development, endocrine active compounds, *in utero* exposure, low-dose effects, male fertility, neurobehavior, PBDE-99

## Abstract

*In utero* exposure to a single low dose of 2,2′,4,4′,5-pentabromodiphenyl ether (PBDE-99) disrupts neurobehavioral development and causes permanent effects on the rat male reproductive system apparent in adulthood. PBDEs, a class of flame retardants, are widely used in every sector of modern life to prevent fire. They are persistent in the environment, and increasing levels of PBDEs have been found in biota and human breast milk. In the present study we assessed the effects of developmental exposure to one of the most persistent PBDE congeners (PBDE-99) on juvenile basal motor activity levels and adult male reproductive health. Wistar rat dams were treated by gavage on gestation day 6 with a single low dose of 60 or 300 μg PBDE-99/kg body weight (bw). In offspring, basal locomotor activity was evaluated on postnatal days 36 and 71, and reproductive performance was assessed in males at adulthood. The exposure to low-dose PBDE-99 during development caused hyperactivity in the offspring at both time points and permanently impaired spermatogenesis by the means of reduced sperm and spermatid counts. The doses used in this study (60 and 300 μg/kg bw) are relevant to human exposure levels, being approximately 6 and 29 times, respectively, higher than the highest level reported in human breast adipose tissue. This is the lowest dose of PBDE reported to date to have an *in vivo* toxic effect in rodents and supports the premise that low-dose studies should be encouraged for hazard identification of persistent environmental pollutants.

Flame retardants have been shown to possess a wide spectrum of toxicity in laboratory animals, and they are present in every level of the food chain. However, this class of substances is important in modern society because of their ability to save lives by limiting the consequences of fires. One class of these chemicals is the polybrominated diphenyl ethers (PBDEs), used in plastics, textiles, foams, and electronic circuitry to avoid fire propagation. They are additives mixed into polymers and, as such, are not chemically bound and can leach into the surrounding environment (de Wit 2002). Because of their high lipophilicity and persistence, PBDEs have been found in sewage sludge, sediment, biota, and humans (Darnerud et al. 2001). Between 1972 and 1997, analysis of human milk has shown a 60-fold increase of PBDE levels in Swedish women (Meironyte et al. 1999), and a recent article has reported much higher levels in human breast adipose tissue in the San Francisco Bay area (She et al. 2002).

PBDEs display a molecular structure similar to that of polychlorinated biphenyls (PCBs), and therefore, one would expect that they also exhibit a different spectrum of toxicity among the 209 possible congeners. PBDE-99 is one of the most persistent congeners detected in almost all environmental samples (Darnerud et al. 2001; de Wit 2002; McDonald 2002). Neurobehavioral toxicity seems to be the most sensitive target of PBDEs in rodents that has been reported to date, although pronounced effects on thyroid homeostasis have been also reported (Hallgren and Darnerud 2002; Hallgren et al. 2001; Zhou et al. 2001, 2002). In rats and mice, pre- and/or postnatal exposure to PBDE-99 has been shown to cause permanent neurobehavioral disturbances in offspring at doses below that able to cause maternal toxicity (Branchi et al. 2002, 2003; Eriksson et al. 2001, 2002; Viberg et al. 2002). One study suggests that cholinergic nicotinic receptors may also be a target for PBDEs, because they found a decrease in α-bungarotoxin binding in hippocampus in mice neonatally exposed to PBDE-153 (Viberg et al. 2003a). However, the mechanism(s) underlying PBDE-induced toxicity is not clear.

There is growing evidence that male reproductive health has been deteriorating over the last few decades. Studies in France, Belgium, Denmark, and Great Britain have reported a significant temporal decline in human semen quality in the last half-century (Auger et al. 1995; Irvine et al. 1996; Van Waeleghem et al. 1996). During the same time, the numbers of hypospadias and cryptorchidisms appear to be increasing (Cour-Palais 1966; Czeizel 1985; Czeizel et al. 1981; Kallen and Winberg 1982; Matlai and Beral 1985; Yucesan et al. 1993), and a similar trend has been observed in the incidence of testicular cancer, which is now the most common malignancy of young men (Adami et al. 1994; Boyle et al. 1987; Forman and Moller 1994; Hakulinen et al. 1986; Nethersell et al. 1984; Pike et al. 1987). Despite the fact that an extensive review of the published data suggests a temporal decline in human sperm production, methodologic bias hinders a final conclusion. The etiology of the malignancies is unknown, but biological plausibility and experimental evidence support the hypothesis that environmental pollutants are acting as endocrine-active compounds. For example, in rodents, reduced sperm counts have been observed at adulthood when animals were pre- and/or postnatally exposed to 2,3,7,8-tetrachlorodibenzo-*p*-dioxin (Faqi et al. 1998b; Theobald and Peterson 1997), PCBs (Faqi et al. 1998a; Hsu et al. 2003; Kuriyama and Chahoud 2004), and the pesticides deltamethrin (Andrade et al. 2002) and lindane (Dalsenter et al. 1997). Nevertheless, there is scant information regarding possible effects of PBDEs on male reproduction.

Because of the increasing levels of PBDEs found in human and biota samples, we conducted the present study to examine the effects of a single low dose of 60 or 300 μg PBDE-99/kg body weight (bw) on gestation day (GD)6 on neurobehavior and male reproductive health in rat offspring. Assuming that fat content in rats is approximately 14% of total body weight, the doses used in this study are approximately 6 and 29 times, respectively, higher than the highest level reported by She et al. (2002; 72.2 μg/kg fat) in human breast adipose tissue It has been demonstrated that PBDE-99 possesses a long half-life (~ 41.6 days in female rat) (Geyer et al. 2004), and we therefore treated the dams on GD6 in order to assess the effects of PBDE-99 during embriofetal and lactational (~ 37 days) periods. Because PBDEs have the ability to interfere with thyroid hormone (TH) homeostasis, we included a reference group for TH-mediated effects by adding the goitrogen 6-*n*-propyl thiouracil (PTU) in the drinking water of pregnant females.

## Material and Methods

### Animals and treatment.

Wistar rats (HsdCpb:WU; Fa. Harlan-Winkelmann, Borchen, Germany) weighing 200 ± 15 g were allowed to acclimatize for 2 weeks. The rats were exposed to constant light/dark periods of 12 hr each, a temperature of 21 ± 1°C, and 50 ± 5% relative humidity. Rodent chow (Altromin 1324; Altromin GmbH, Lage, Germany) and tap water were available *ad libitum*. Two nongravid females were placed with one male for 3 hr, and the day of sperm detection in the vaginal smear was considered day 0 of gestation. The gravid females were randomly assigned among the four groups and housed individually in type III Macrolon cages with stainless steel covers and wood shavings (Altromin GmbH). 2,2′,4,4′,5-Pentabromodiphenyl ether (PBDE-99; 98% pure), lot number VL02, was purchased from LGC Promochem GmbH (Wesel, Germany). Pregnant rats were treated orally by gavage with a single dose of 60 μg PBDE/kg (*n* = 20) or 300 μg PBDE-99/kg (*n* = 19) on GD6. The control pregnant rats (*n* = 16) received the vehicle, peanut oil, in a volume of 10 mL/kg bw on the same day. An additional group was administered the goitrogen PTU (6-n-propyl-2-thiouracil; Sigma-Aldrich Chemicals GmbH, Steinheim, Germany), which served as a reference control for TH effects. PTU was given to the gravid dams by adding 5 mg/L PTU in the drinking water on GD7–21. Dams were allowed to deliver, and the litter size was not artificially altered. The experimental protocol has the approval of the National Animal Protection Law (Tierschutzgesetz BGBI. IS. 3082, 2002).

### Postnatal reflex and developmental landmarks.

Developmental landmarks (eruption of incisors, fur development, eye opening, and testes descent) and postnatal reflexes were evaluated in all pups (control, *n* = 163; PTU, *n* = 200; 60 μg PBDE, *n* = 218; 300 μg PBDE, *n* = 200). Starting on postnatal day (PND)3, we monitored the offspring for the development of spontaneous cliff-drop aversion reflex, and beginning on PND18, we examined their ability to stay on a rotating rod for 3 min at 7 rpm.

### Locomotor activity.

Circadian motility was measured over 24-hr periods on PND36 and PND71 in individual offspring using a Mobilitron (FU-Berlin/Eisenberger GmbH, Dillenburg, Germany), a device that monitors the locomotion of the animal at 5-min intervals using three infrared photocells per cage. Habituation in the Mobilitron took place for a 24-hr period before testing began in order to allow the animals to adjust to their new environment and the solitary accommodation before measurements were taken. The locomotor activity of one male and one female per litter per group (one animal per cage) were evaluated before puberty (PND36) and after puberty (PND71). The animals were randomly assigned in the Mobilitron (which allows simultaneous measurement of 48 cages) to avoid confounding factors. The method has been described in detail by Thiel et al. (1989).

### Reproductive assessment of adult male offspring.

At adulthood (~ PND140), 12 males/group (from different litters) were killed by decapitation. Trunk blood was collected for hormone analysis, and organ weights (thymus, spleen, liver, testis, epididymis, seminal vesicle, and ventral prostate) were recorded. The right testis and caudal epididymis were kept in saline buffer for spermatid and sperm counts, respectively.

### Spermatid number.

The testis was minced and homogenized for 1 min in 10 mL 0.9% NaCl containing 0.5% Triton X-100 at medium speed in an IKA-RW 15 Tissuemizer (Janke and Kunkel, Staufen in Breisgau, Germany). The number of homogenization-resistant spermatids was counted in a Buerker hemocytometer (Brand GmbH, Wertheim, Germany). Daily sperm production was calculated, dividing the number of homogenization-resistant spermatids by 6.1 (Robb et al. 1978).

### Sperm count and morphology.

Cauda epididymis was minced and homogenized for 1 min in 10 mL 0.9% NaCl containing 0.5% Triton X-100 at medium speed in an IKA-RW 15 Tissuemizer. The number of homogenization-resistant sperm was counted in a hemocytometer (Buerker). For sperm morphology, the ductus deferens was rinsed with 1 mL 0.9% NaCl to obtain a sperm suspension. Aliquots were stained with 2% eosin to assess the percentage of morphologically abnormal sperm by evaluating 200 sperm/animal.

### Testosterone and luteinizing hormone levels.

After decapitation, trunk blood was collected and allowed to clot on an ice bath (4°C) for 2 hr. Serum was collected via centrifugation of clotted samples (2,500 rpm for 15 min) and stored at –20°C for later analyses. Serum testosterone and luteinizing hormone (LH) were measured using the ELISA kit purchased from DRG Diagnostics GmbH (Marburg, Germany). Testosterone was measured in crude rat serum, which is reliable for comparison among groups, but matrix effects cause uncertainties with respect to absolute values.

### Male reproductive performance.

Adult male offspring (± 150 days of age; *n* = 15–19 animals/group), representing all litters, were mated with untreated females (1:1) daily for 14 days to determine whether the males were fertile and could sire normal offspring. Vaginal smears were collected daily and examined for the presence of sperm. The day of sperm detection in vaginal smears was considered day 0. The dams were sacrificed on GD21 and the uterus was excised. The uterine and fetal weights and the numbers of implantations, resorptions, and fetuses were determined. The fetuses were examined for external anomalies and sexed.

### Male sexual behavior.

Approximately on PND160, 20 males/group (representing all litters) were mated with untreated females in estrus (1:1), and the sexual behavior of each mating was recorded for 20 min under blue light illumination (black light, 75W; Osram, Berlin, Germany) using a video camcorder (Hi8 Handycam CCD-V800E, Sony, Tokyo, Japan). The recorded videos, which provide a permanent record and the opportunity for replay, were evaluated by a trained observer in a blind fashion. The phase of the estrous cycle of the untreated females was predetermined by examining vaginal smears. The method was previously described in detail by Chahoud and Faqi (1998).

### Statistical analyses.

The statistical analyses were performed with SPSS software, version 11.5 for Windows (SPSS Inc., Chicago, IL, USA). Data for males and females within each group were tested by the Student *t*-test. Data with normal distribution were analyzed by analysis of variance (ANOVA) followed by the Dunnett *t*-test. The equality of survival distributions was examined using the Kruskal-Wallis test followed by the Mann-Whitney *U* test. Proportions were analyzed by Fisher’s exact test, and statistical differences were considered significant when *p* < 0.05.

## Results

### Spontaneous behavior and developmental landmarks.

**Figure 1:**
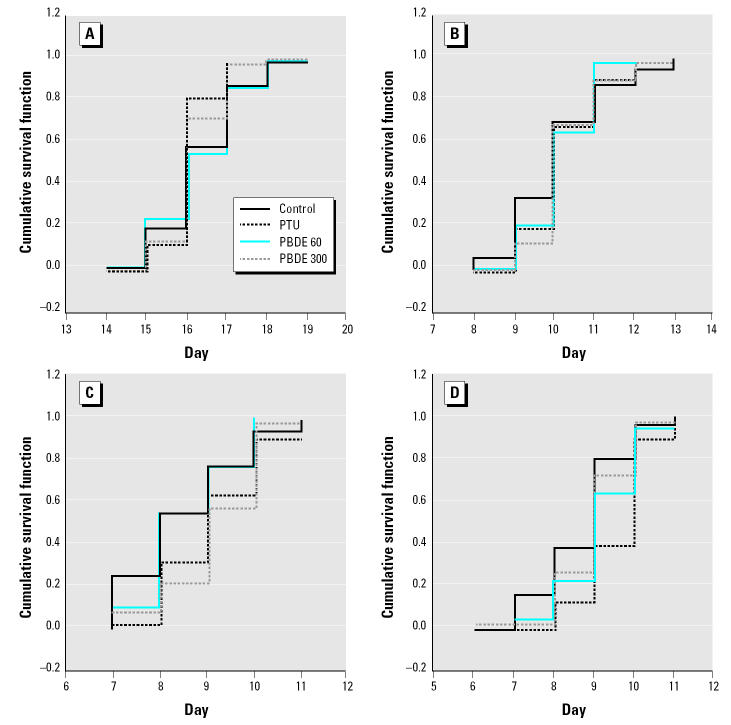
Cumulative survival function of the spontaneous reflex, cliff-drop aversion, and the developmental landmarks eye opening and eruption of incisors from rat offspring after pre- and postnatal (via milk) low-dose PBDE-99 (60 or 300 μg/kg bw) exposure. (*A*) Time of eye opening. (*B*) Time of eruption of incisors. (*C*) Time to develop the cliff-drop aversion reflex in male offspring. (*D*) Time to develop the cliff-drop aversion reflex in female offspring. Groups were compared using the Kruskal-Wallis test followed by the Mann-Whitney *U* test, and differences were considered statistically significant when *p* < 0.05: PTU in (*A*) and (*D*); PTU and PBDE 300 in (*B*) and (*C*).

Spontaneous behavior and developmental landmarks are shown in Figure 1. Except for cliff-drop aversion reflex, no differences between sexes were detected in the statistical analysis, and therefore, the male and female data were pooled for the analysis of all the developmental landmarks and the rotating rod reflex. The age at fur development, testes descent, and the ability to master the rotating rod test were not different between treated and control animals (data not shown). However, cumulative survival function analysis for the age at eye opening, eruption of incisors, and the cliff-drop aversion reflex revealed a statistically significant difference among the groups. The onset of eye opening was earlier in the PTU-treated litters than among controls (Figure 1A), and the eruption of incisors was delayed in the groups treated with PTU or 300 μg/kg PBDE compared with controls (Figure 1B). The development of the cliff-drop aversion reflex was significantly delayed in both PTU-exposed male and female offspring as well as in males exposed to the 300 μg dose of PBDE-99 (Figure 1C,D).

### Locomotor activity.

**Figure 2:**
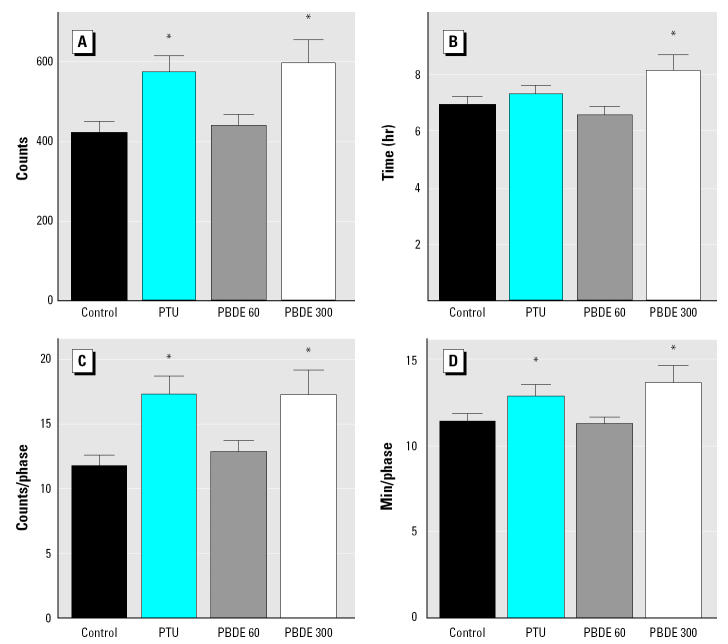
Locomotor activity of rat offspring after pre- and postnatal (via milk) low-dose PBDE-99 (60 or 300 μg/kg bw) exposure: quantitative and qualitative analysis on PND36 showing total activity (LBI) and duration of activity per day and per active phase. (*A*) LBI counts per day. (*B*) Duration (hours) of activity per day. (*C*) LBI counts per active phase. (*D*) Duration of activity (minutes) per active phase. Bars represent mean ± SEM.
*p < 0.05; significances were detected by ANOVA, followed by the Dunnett *t*-test when *p* < 0.05.

**Figure 3:**
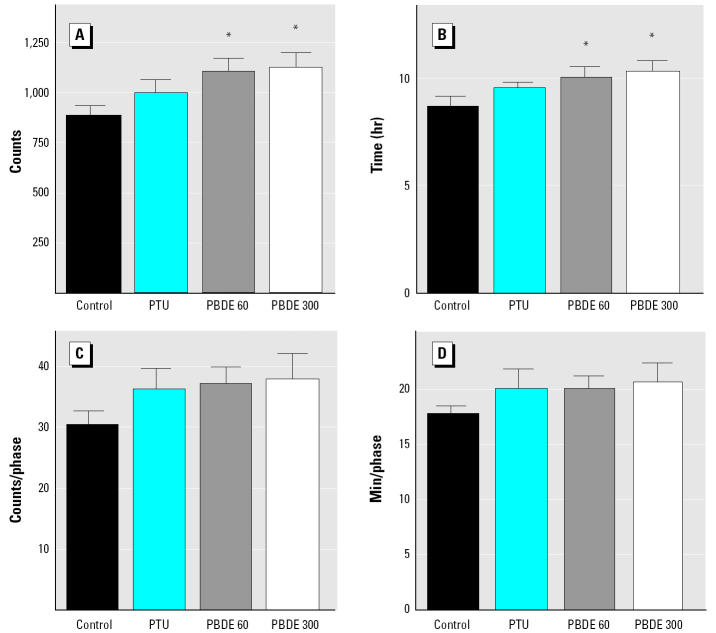
Locomotor activity of rat offspring after pre- and postnatal (via milk) low-dose PBDE-99 (60 or 300 μg/kg bw) exposure: quantitative and qualitative analysis on PND71 showing total activity (LBI) and duration of activity per day and per active phase. (*A*) LBI counts per day. (*B*) Duration (hours) of activity per day. (*C*) LBI counts per active phase. (*D*) Duration of activity (minutes) per active phase. Bars represent mean ± SEM.
*p < 0.05; significances were detected by ANOVA, followed by the Dunnett *t*-test when *p* < 0.05.

Using the Mobilitron apparatus, individual locomotion of rats was measured over 24 hr in young and pubertal offspring. Statistical analysis revealed no difference between the sexes for all groups tested, and therefore, the data from the males and females are presented together. On PND36, the total light beam interruption (LBI) count per day was significantly greater in the PTU and 300 μg/kg PBDE groups (Figure 2A). The number of active hours per day was longer in the PBDE 300 μg/kg group, an effect not seen in the PTU group (Figure 2B). The qualitative analysis (i.e., LBI count per phase and duration of activity per phase) on the same day (PND36) confirms what was observed in the quantitative analysis. Both the PTU and 300 μg/kg PBDE groups were more active during the active phases compared with control, and the duration of the active phases were also longer even though there were no statistically significant differences in the number of active phases (Figure 2C,D). An active phase is defined when the animal begins to move (associated with LBIs) until a pause (a period of no LBI) is observed. No differences compared with control were seen in the 60 μg PBDE group on PND36. At puberty (PND71), the quantitative analysis indicated that the two PBDE groups were hyperactive compared with controls. In other words, both the LBI counts and duration of activity per day were significantly increased in 60 μg PBDE and 300 μg PBDE groups. In that age (PND71), no statistically significant qualitative differences were observed among the groups (Figure 3).

### Body and organ weights of adult male offspring (PND140).

Body, liver, thymus, and spleen weights of adult male offspring are given in Table 1. We observed no changes in body, liver, and thymus weights related to the treatment. Pre- and postnatal exposure to PBDE-99 and gestational exposure to PTU produced a significant increase in absolute spleen weight (Table 1). However, when spleen weight was expressed as a ratio of body weight (relative weight), only animals in the 60 μg PBDE-99 group exhibited the same trend (Table 1).

### Male fertility and reproductive performance.

Table 2 shows the reproductive organ weights, as well as sperm and spermatid counts, sperm morphology, and steroid hormone levels. We found no significant differences in the absolute testis and epididymis weights; however, when expressed as a percentage of body weight (relative weight), the PTU and PBDE 300 μg groups had smaller testes, whereas the epididymis relative weights were decreased in all three treatment groups compared with controls. No differences were observed in prostate and seminal vesicle (absolute and relative) weights (Table 2). The lower testis and epididymis weights were accompanied by reductions in sperm and spermatid counts as well as daily sperm production. Reductions in testicular spermatid count and sperm count from caudal epididymis were observed in all treatment groups (Table 2). It is noteworthy that daily sperm production was reduced by approximately 30% from controls. The decrease in sperm production was not associated with poor sperm quality because the percentage of abnormal sperm was within normal limits in all groups. Testosterone and LH levels were also not affected, suggesting a minor role for steroid hormones in the impairment of sperm production. When the litter mates of the animals analyzed for sperm counts were mated with untreated females for fertility studies, exposed males could sire offspring similar to the control males (Table 3). Uterine weight, litter size, and numbers of implantations, resorptions, and viable fetuses were within the normal range of control (Table 3).

### Sexual behavior.

Pre- and postnatal exposure to PTU or either dose of PBDE-99 did not impair sexual behavior of the adult male offspring. Ejaculatory and mounting latencies, intromission frequency and latency, and number of penetrations were normal when all groups were compared with controls (Table 4). However, the number of animals that had two or more ejaculations during 20 min of mating was significantly lower in the PBDE-exposed animals. Approximately 50% of controls had a second ejaculation, whereas only 39% and 21% of the males from the 60 μg PBDE and 300 μg PBDE groups, respectively, achieved a second ejaculation (Table 4).

## Discussion

In the present study we found consistent evidence that exposure to low doses of PBDE-99 during critical periods of development affects motor activity and permanently impairs spermatogenesis in adult rat offspring. This is the lowest dose of PBDE reported to date to have an *in vivo* toxic effect in rodents. We observed neurobehavioral changes on PND36 and PND71 in offspring exposed to the highest dose (300 μg/kg bw), whereas behavioral changes were present only at puberty in the lowest dose tested (60 μg/kg bw; Figures 2 and 3). Other groups have reported neurodevelopmental disturbances in rodents exposed to PBDEs (Branchi et al. 2002, 2003; Eriksson et al. 2001; Viberg et al. 2003a, 2003b), and this system seems to be the most sensitive to PBDE-induced toxicity. Supporting our data, Eriksson et al. (2001) found that neonatal exposure (single dose on PND10) to PBDE-99 or PBDE-47 disrupts spontaneous behavior and causes hyperactivity in mice that appears to be permanent and to worsen with age (Eriksson et al. 2001). The crucial role of THs during brain development is well known, and disturbances in TH homeostasis (e.g., PTU exposure) can cause serious impairment in neurologic development (Porterfield 1994). In support of our findings, previous studies have reported hyperactivity in rodent offspring after pre- and postnatal hypothyroidism induced by goitrogens such as PTU (Akaike et al. 1991; Davenport and Hennies 1976; Goldey et al. 1995; Tamasy et al. 1986). However, the behavioral changes in PBDE-99 groups (persistent at least until PND71) observed in this study are not similar to our reference group for TH-mediated effects (PTU; transient hyperactivity only on PND36), suggesting that the neurotoxicity induced by PBDE-99 may stem from mechanism(s) other than those caused by PTU. Even though more mechanistic studies are lacking, the cholinergic system seems to be affected after neonatal exposure to PBDE, because Viberg et al. (2003a) found reduced amounts of nicotinic receptors in the hippocampus of exposed animals using an α-bungarotoxin assay, and the response of the cholinergic agent nicotine was altered in mice neonatally exposed to PBDE-99 (Viberg et al. 2002, 2003a). In that way, hyperactivity induced by PBDE-99 might be explained by changes in the cholinergic system during pre-and postnatal exposure. Nevertheless, one should not rule out other mechanisms because some hydroxylated PBDE metabolites have been shown to possess high binding affinities to TH receptors (Marsh et al. 1998). It is plausible to hypothesize that also the binding of the PBDE-99 molecule or its metabolite to the TH receptor in the developing brain could cause neurobehavioral disturbance in offspring.

Increasing evidence suggests that continuous exposure to environmental pollutants is related to the postulated deterioration of male reproductive health in the last 50 years. This hypothesis highlights the need for more experimental studies that employ doses relevant to environmental/human exposure scenarios in order to elucidate possible mechanisms involved in such a decline. In this study, developmental exposure to low-dose PBDE-99 not only caused persistent neurobehavioral effects but also permanently affected adult male reproductive health (Table 2). This is the first report on effects of PBDE-99 on male reproductive performance because our survey of the literature failed to find data on this topic. Questions regarding persistent chemical contamination typically focus on bioaccumulation, neurotoxicity, and carcinogenicity. However, the male reproductive system has been shown to be a very sensitive end point when the insult occurs during critical periods of development (Andrade et al. 2002; Cooke et al. 1992; Dalsenter et al. 1997; Faqi et al. 1998b; Kuriyama and Chahoud 2004; Sharpe et al. 1995). In the mating study, no effect on fertility was seen when males were mated with untreated females, which is not inconsistent with the observed decrease in daily sperm count of the littermates (Tables 2 and 3). In rats, sperm production can be reduced up to 90% without compromising fertility (Aafjes et al. 1980; Kirby et al. 1992). On the other hand, relatively small changes in sperm production in men may have severe consequences for human reproduction (Zenick and Clegg 1989). Because the normal human sperm count is near the threshold for the number of sperm needed to ensure reproductive competence, sperm count is a sensitive and validated end point for reproductive toxicology assessment. The growth and maturation of the developing testis as well as the maintenance of spermatogenesis are regulated by several endocrine and paracrine factors. Among them, thyroid function during early life has a major impact on regulating testicular growth and function. When rats are made hypothyroid during a critical window of neonatal development, permanent increases in adult testis size and sperm production have been observed (Cooke et al. 1992). However, this effect occurs only when rats are hypothyroid during the first week of postnatal development (Cooke et al. 1992). Using a dose 200-fold lower than that reported by Cooke et al. (1992), we observed that prenatal hypothyroidism induced by PTU caused an opposite effect, namely, decreased sperm production and testis size. Impaired spermatogenesis and reduced testicular weight seen in males exposed to both doses of PBDE-99 might also be correlated to alterations in TH concentrations. However, the mechanisms underlying these effects observed both in PTU-exposed and PBDE-99–exposed animals remain to be elucidated. This study demonstrates for the first time that exposure to a low dose of PBDE-99, which resembles the human exposure levels, causes permanent impairment of spermatogenesis in rats. These findings encourage further investigation on mechanistic studies in order to assess the hazard of flame retardants on human reproductive health. Moreover, the issue of synergistic or additive effects when PBDEs are combined to other persistent pollutants (e.g., PCBs and DDT) remains to be elucidated.

## Figures and Tables

**Table 1:** Absolute and relative (percent of body weight) organ weights from adult offspring (PND140) exposed pre- and postnatally (via milk) to PBDE-99 (*n* = 12/group).

			PBDE	
Parameters	Control	PTU	60 μg/kg bw	300 μg/kg bw
Body weight (g)	311.7 ± 8.3	335.9 ± 9.9	320.5 ± 5.9	334.9 ± 8.6
Liver weight (g)	10.43 ± 0.47	11.17 ± 0.53	10.82 ± 0.21	11.26 ± 0.39
Percent bw	3.35 ± 0.08	3.31 ± 0.07	3.38 ± 0.04	3.36 ± 0.07
Thymus weight (g)	0.34 ± 0.02	0.34 ± 0.03	0.36 ± 0.02	0.32 ± 0.03
Percent bw	0.11 ± 0.006	0.10 ± 0.008	0.11 ± 0.005	0.10 ± 0.007
Spleen weight (g)	0.55 ± 0.01	0.63 ± 0.03*	0.60 ± 0.02*	0.60 ± 0.02*
Percent bw	0.17 ± 0.004	0.19 ± 0.006	0.19 ± 0.005*	0.18 ± 0.004

Absolute and relative organ weights were analyzed using ANOVA followed by the Dunnett *t*-test. Values are presented as mean ± SEM.

* *p* < 0.05.

**Table 2:** Reproductive organ weights, hormone levels, sperm number, and daily sperm production in adult offspring (PND140) exposed pre- and postnatally (via milk) to PBDE-99 (*n* = 12/group).

			PBDE	
Parameters	Control	PTU	60 μg/kg bw	300 μg/kg bw
Testis weight (g)	1.57 ± 0.06	1.47 ± 0.10	1.58 ± 0.03	1.53 ± 0.04
Percent bw	0.51 ± 0.02	0.44 ± 0.03*	0.49 ± 0.01	0.46 ± 0.01*
Epididymis weight	0.58 ± 0.02	0.55 ± 0.02	0.56 ± 0.01	0.58 ± 0.02
Percent bw	0.19 ± 0.01	0.17 ± 0.01*	0.18 ± 0.002*	0.17 ± 0.007*
Seminal vesicle weight empty (g)	0.99 ± 0.04	1.11 ± 0.04	1.00 ± 0.03	1.04 ± 0.05
Percent bw	0.32 ± 0.01	0.33 ± 0.01	0.31 ± 0.01	0.31 ± 0.01
Prostate (g)	0.38 ± 0.03	0.40 ± 0.02	0.40 ± 0.01	0.43 ± 0.03
Percent bw	0.12 ± 0.01	0.12 ± 0.01	0.12 ± 0.003	0.13 ± 0.008
Spermatid (10^6^)	266.2 ± 7.5	198.6 ± 10.5*	182.8 ± 7.6*	175.0 ± 5.7*
Daily sperm production (10^6^)	43.6 ± 1.2	32.6 ± 1.7*	30.0 ± 1.2*	28.7 ± 0.9*
Sperm number (10^6^)	189.6 ± 11.7	143.2 ± 8.0*	134.7 ± 6.4*	156.3 ± 8.1*
Abnormal sperm (%)	6.3 ± 0.8	7.7 ± 1.0	5.6 ± 0.5	7.9 ± 1.0
LH (ng/mL)	10.8 ± 1.2	12.4 ± 1.3	14.4 ± 2.1	10.3 ± 1.1
Testosterone (ng/mL)	8.7 ± 1.2	10.0 ± 1.4	7.5 ± 1.0	8.4 ± 1.4

Absolute and relative organ weights were analyzed using ANOVA followed by the Dunnett *t*-test. Values are presented as mean ± SEM.

* *p* < 0.05.

**Table 3:** Reproductive performance of adult male offspring exposed pre- and postnatally (via milk) to PBDE-99.

			PBDE	
Parameters	Control	PTU	60 μg/kg bw	300 μg/kg bw
No. of dams	19	18	15	19
Body weight gain (%)	49.3	46.3	47.5	50.9
Uterine weight (g)	73.6 ± 4.3	77.8 ± 1.7	71.2 ± 2.7	71.1 ± 3.5
Implantations (*n*)	214	203	161	217
Implantations/litter	11.3 ± 0.18	11.3 ± 0.11	10.7 ± 0.15	11.4 ± 0.12
Viable fetuses/litter	10.8 ± 0.19	10.9 ± 0.11	10.1 ± 0.15	10.3 ± 0.17
Total resorptions (%)	9 (4)	6 (3)	10 (6)	21 (10)
Fetal weight/litter (g)	4.70 ± 0.10	4.66 ± 0.06	4.96 ± 0.13	4.85 ± 0.07
Sex ratio (male/female)	47.3/52.7	47.4/52.6	46.4/53.6	42.9/57.1

Male rats were pre- and postnatally exposed to a low dose (60 μg or 300 μg/kg bw) of PBDE-99 and mated with non-exposed females. Values are presented as mean ± SEM.

**Table 4:** Male sexual behavior of adult offspring exposed pre- and postnatally (via milk) to PBDE-99.

Parameters	Control	PTU	PBDE 60	PBDE 300
No. of animals with ejaculation/total no. (%)	17/20 (85)	18/20 (90)	19/20 (95)	17/20 (85)
Mounting latency (sec)	24.7 ± 3.9	23.4 ± 4.5	41.0 ± 9.6	27.2 ± 4.9
Intromission latency (sec)	50.7 ± 11.6	37.2 ± 7.3	54.7 ± 10.1	35.1 ± 5.5
Ejaculatory latency (min)	11.1 ± 0.7	8.4 ± 1.0	12.9 ± 1.1	12.5 ± 0.8
Intromission frequency (*n*/min)	0.99 ± 0.08	0.97 ± 0.12	1.06 ± 0.08	1.23 ± 0.10
No. of penetrations before the first ejaculation	19.8 ± 1.5	19.5 ± 2.5	21.3 ± 1.5	24.7 ± 2.0
Percent of animals with two or more ejaculations	53	71	39	21*

Animals were pre- and postnatally exposed to a low dose of PBDE-99 (60 μg or 300 μg/kg bw) or PTU (5 mg/L). Adult offspring were mated with nonexposed females. Values are presented as mean ± SEM.

* *p* < 0.05.
